# Using email reminders to engage physicians in an Internet-based CME intervention

**DOI:** 10.1186/1472-6920-4-17

**Published:** 2004-09-29

**Authors:** Maziar Abdolrasulnia, Blanche C Collins, Linda Casebeer, Terry Wall, Claire Spettell, Midge N Ray, Norman W Weissman, Jeroan J Allison

**Affiliations:** 1 Division of Continuing Medical Education, University of Alabama at Birmingham, JNWB 406, 1530 3^rd ^Avenue South, Birmingham, AL 35294-0500, USA; 2 Center for Outcomes and Effectiveness Research Education, University of Alabama at Birmingham, MT 401, 1530 3^rd ^Avenue South, Birmingham, AL 35294-1170, USA; 3 Department of Pediatrics, University of Alabama at Birmingham, MTC 201 1600 7^th ^Avenue South 35294-0011, USA; 4 Aetna Integrated Informatics, Inc. 151 Farmington Avenue Hartford, CT 06156, USA; 5 School of Health Related Professions, University of Alabama at Birmingham, Webb Building 564, 1530 3^rd ^Avenue South, Birmingham, AL 35294-3361, USA; 6 Division of General Medicine, University of Alabama at Birmingham, FOT 720D 20^th ^Street South, Birmingham, AL 35294-3407, USA

## Abstract

**Background:**

Engaging practicing physicians in educational strategies that reinforce guideline adoption and improve the quality of healthcare may be difficult. Push technologies such as email offer new opportunities to engage physicians in online educational reinforcing strategies. The objectives are to investigate 1) the effectiveness of email announcements in engaging recruited community-based primary care physicians in an online guideline reinforcement strategy designed to promote Chlamydia screening, 2) the characteristics of physicians who respond to email announcements, as well as 3) how quickly and when they respond to email announcements.

**Methods:**

Over a 45-week period, 445 recruited physicians received up to 33 email contacts announcing and reminding them of an online women's health guideline reinforcing CME activity. Participation was defined as physician log-on at least once to the website. Data were analyzed to determine participation, to compare characteristics of participants with recruited physicians who did not participate, and to determine at what point and when participants logged on.

**Results:**

Of 445 recruited physicians with accurate email addresses, 47.2% logged on and completed at least one module. There were no significant differences by age, race, or specialty between participants and non-participants. Female physicians, US medical graduates and MDs had higher participation rates than male physicians, international medical graduates and DOs. Physicians with higher baseline screening rates were significantly more likely to log on to the course. The first 10 emails were the most effective in engaging community-based physicians to complete the intervention. Physicians were more likely to log on in the afternoon and evening and on Monday or Thursday.

**Conclusions:**

Email course reminders may enhance recruitment of physicians to interventions designed to reinforce guideline adoption; physicians' response to email reminders may vary by gender, degree, and country of medical training. Repetition of email communications contributes to physician online participation.

## Background

Many clinical practice guidelines have been disseminated through print publications and the Internet to improve the quality of healthcare. Complex physician behavior-change interventions have been created and applied to clinical practice guidelines to determine if the guidelines are being practiced. Research indicates that the adoption of clinical practice guidelines continues to lag behind their production and dissemination [1]. Adoption of such guidelines has been improved by the use of secondary strategies, which involve education, audit and feedback, benchmarking, office system interventions, and multifaceted interventions [1]. Continuing medical education (CME) providers and others interested in improving the quality of health care have had difficulty actively engaging practicing physicians in secondary strategies that may lead to guideline adoption [2], since CME courses and other educational interventions must compete with the physician learner's multiple priorities [3].

Over the past ten years, accessibility to educational opportunities has increased with advancements in technology, particularly the Internet. The Internet offers a delivery system for educational interventions and research activities that are more convenient to the physician learner than traditional live large group lectures and seminars [4]. The Internet generally functions in a passive manner, or as a "pull" technology, allowing the user to determine when, where and how to seek information. Engaging physicians in online educational activities may require the use of "push" technologies rather than pull technologies. "Push" technologies allow information to be delivered to the user rather than requiring the user to actively search for the desired information; they require minimal effort on the part of the recipient, which greatly supports their adoption [5]. Email was the first type of online push technology [5]. Other forms of push technologies are actively used such as pop-ups, list-serves, and screen savers. Based on preliminary research presented at the ***Proceedings of the American Medical Informatics Association's 2002 Annual Symposium***, screen savers have been shown to be effective as a reminder system in engaging physicians in bioterrorism CME activity, although the use of email has exceeded all other Internet applications [5].

Flanagan and colleagues demonstrated promise for email as a means of engaging physicians in online activities designed to reinforce guideline use. They found physician response rate to email solicitation to be 50% over a 14-month study period [6]. Beyond this, little is known about the effectiveness of email in engaging physicians in online educational interventions designed to improve the quality of healthcare [7]. The purposes of this study were to investigate 1) the effectiveness of email reminders in engaging recruited community-based primary care physicians in an online guideline reinforcement strategy that was designed to promote women's health, 2) the characteristics of physicians who respond to email announcements, as well as 3) how quickly and when they respond to email announcements.

## Methods

This study is a subanalysis of data from a randomized controlled trial (RCT), described in detail elsewhere [8]. The goal of the RCT was to improve Chlamydia screening rates of at-risk young women by community-based primary care physicians by using a multi-faceted online guideline reinforcement intervention. Primary care physicians were recruited from a national sample of 923 eligible offices identified by a large managed care organization's administrative data. Eligible offices had at least 20 young women (ages 16–26) at risk for chlamydial infection and at least one primary care physician (Internal Medicine, Family Medicine/General Practice, Pediatrics) with Internet access. In Phase I of recruitment occurred at the office level and Phase II at the physician level. In Phase I, all eligible offices were invited to participate via facsimile; an office was designated as "recruited" when one of its physicians declared intent to participate. In Phase II, an active link to the Intervention module was e-mailed to physicians recruited in Phase I. Recruited physicians were assigned at the time of logon to either a control or Internet CME intervention arm designed to improve Chlamydia screening rates; physicians within the same office were assigned to the same arm of the study. We designated physicians as "participating" when they first engaged the Internet intervention.

The study was conducted between February 1, 2002 and December 31, 2002. The intervention included a series of four modules, feedback of performance data and a quality improvement toolbox. The education modules were based upon adult learning and behavior change theory [8]. Our driving principles included case-based learning [9,10] making programs interactive by adapting to the program learners' readiness-to-change stage [11] and performance feedback for behavioral motivation and reinforcement [12] The control condition was a series of four text-based modules on topics unrelated to Chlamydia. Physicians receive 1 category 1 CME credit per module for their participation. The main outcome measure was Chlamydia screening rates.

The subanalysis of this trial focuses on the use of email reminders to engage physicians in the online intervention. Physician recruitment occurred from November of 2001 to January of 2002. Following recruitment, the intervention was initiated in February of 2002 via email broadcast to recruited physicians. During 2002, four separate educational modules were offered. Each module was introduced with a series of email announcements followed by email reminders that contained tailored subject line (i.e. Dear Dr. John Smith) and text message containing the URL that would connect the physician directly to the educational module. The first three modules, emphasized: (1) young sexually active women are at high risk for asymptomatic infection that may lead to future serious health consequences; (2) newer urine-based screening allows diagnosis without a pelvic examination; and (3) infection may be effectively treated with a one-dose antibiotic. The fourth module reviewed previously introduced concepts. A total of 33 announcements and reminders were sent to recruited physicians between February 1, 2002 and December 31, 2002, which represents one reminder overall for each 1.5 weeks of the study duration. Initially, we were concerned that frequent email reminders would be considered intrusive. After a trial period, we found that we could increase the frequency of reminders without difficulty, thus fewer email reminders were sent during the initial phases of the study.

For the subanalysis of this trial, the principal outcome measure is participation, defined as physician log-on at least once to the website. Data was collected electronically when physicians logged on to the intervention. In addition, several evaluation questions were used at the conclusion of the online educational activities to explore CME preferences. Descriptive analyses of patient and physician demographics are used to compare baseline characteristics of patients and physicians in the email participants versus the recruited non-participants. Statistical significance is determined with tests appropriate to the distribution of the data (chi-square for categorical variables and student-t test for continuous variable). Two-tailed tests are used for all analyses.

## Results and discussion

Four hundred eighty physicians were recruited to participate, representing 380 physician offices. Of the 463 recruited physicians, 445 were successfully contacted by email using the addresses they had furnished at the time of recruitment. Of these 445 recruited physicians, 210 (47.2%) physicians from 190 offices logged on to at least one of the educational modules. Of the 210 physicians who logged on at least once during the 45-week study period, one hundred twenty-four (59%) returned again to log-on for Module 2, eighty-seven (41%) logged on for Module 3, and forty-four (21%) logged on to Module 4.

**Figure 1:**
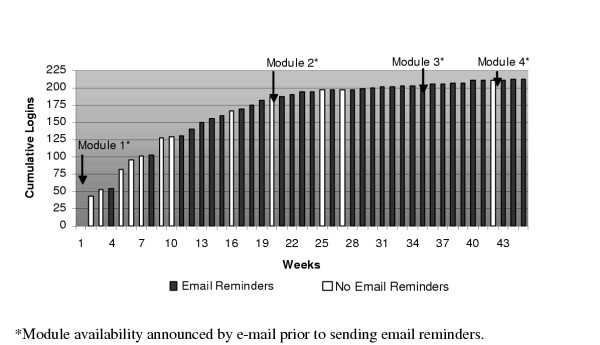
Cumulative internet engagement by physician over time

**Figure 2:**
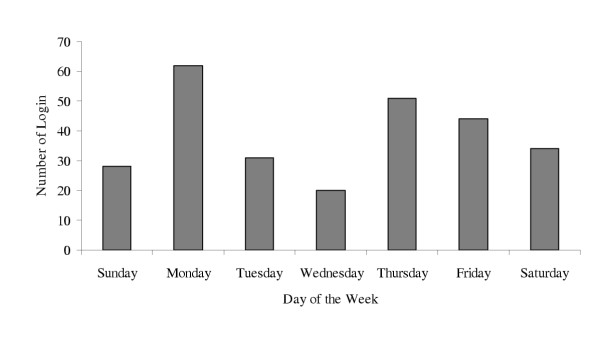
Number of logins by day of the week.

**Figure 3:**
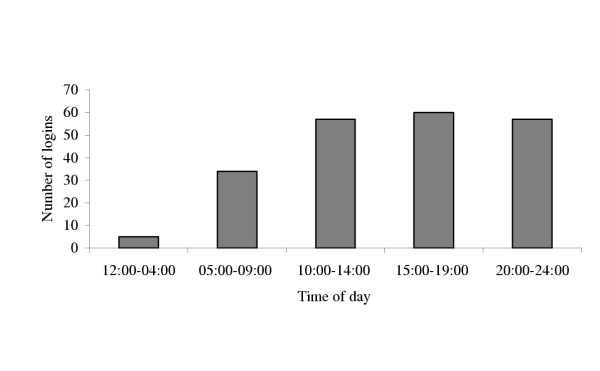
Number of logins by time of day.

Two hundred and ten physicians of 445 logged on at least once to the website, leaving 235 physicians as non-participants. Figure 1 represents total physician log-on by week. Analysis of log-on days indicated that participants were most likely to log-on on Monday or Thursday (see Figure 2). Log-on times were also examined and findings indicate that physicians were most likely to log-on to a module between the hours of 3 P.M. and 7 P.M (15:00–19:00). Other common times for participants to log-on included times earlier in the day, or between the hours of 8 P.M. and midnight (20:00–24:00) (see Figure 3).

The participant characteristics (n = 210) were compared with those of recruited but non-participating physicians (n = 235) (Table 1). Age was not significant, however there were significant differences by gender, degree, and country of medical training. By race, 81.5% of the participants were Caucasian, 8.8% were Asian, 3.0% were African American, 3.5% were Hispanic and 3.0% were listed as other with no significant differences. The largest percentage of recruited physicians was family practitioners (41.3%), followed by general internists (29.4%), pediatricians (9.6%), and general practitioners (1.5%), with no significant differences. Female physicians were significantly more likely to participate than males (p = . 0001), Medical Doctors (MDs) were significantly more likely than Doctors of Osteopathy (DOs) to participate (p = . 01), and graduates of U.S. medical schools significantly more likely than graduates of international medical schools to participate (p = . 01). In addition to demographic characteristics, chlamydia screening rates of participant and non-participant physicians were compared. Baseline screening rates of nonparticipants were significantly lower than those of participants. Non-participant chlamydia screening rates were 14.6% at baseline, compared to 17.4% for participants (p < .006).

**Table 1:** Participant* versus recruited** but non-participant characteristics

	Recruited Non-participants	Participants	p-value
N	235	210	
Mean Age	44.3	45.2	.143
Gender			
Male	60.0%	40.0%	.0001
Female	40.0%	60.0%	
Degree			
DO	65.3%	34.7%	.01
MD	51.9%	48.0%	
Medical Training			
International	67.5%	32.5%	.01
USA	52.7%	47.3%	

*Participated physician engaged in at least one module

**Eligible offices had at least 1 eligible physicians with at least 20 female patients who were candidates for Chlamydia screening according to the Health Employers Data and Information Set Technical Specifications, 2000.

From follow-up evaluation question data, 100% of responding DOs felt that the course email reminders were effective in reminding them about educational modules compared to 92.6% of MDs; 95.9% of US medical graduates rated the reminders as useful, while only 77.8% of graduates from non-US medical schools found them useful. Concerning preferences for delivery of CME, no DOs reported web-based activities as their preferred method for lifelong learning; they preferred local (50.0%) and national meetings (50.0%). MDs were more likely to prefer web-based CME (37.0%) to local (35.1%) or national (16.6%) meetings. Female physicians reported a preference for web-based CME (50.0%), whereas male physicians preferred local (35.0%) and national (27.5%) meetings over web-based CME (25.0%).

The results of this study indicate that email reminders may be useful in engaging nearly half of a group of practicing primary care physicians recruited to participate in an online women's health educational series. This data is consistent with McMahon et al.'s findings in comparing the use of email, fax and mail, finding that email reminders were more useful to increase response rates [12]. The study participation rate, (47.2%) is also consistent with the work of Flanagan et. al.'s study of participation in web decision support tools for the management of pneumonia [6]. However, the gap of up to 3 months between recruitment and the initiation of the online educational activities may have contributed to a lower participation rate. It is possible that by decreasing the gap in time between recruitment and announcement of the availability of the online educational activity, participation rates could be increased. Future study designs using email reminders should consider beginning the intervention immediately following agreement to participate or shortly thereafter. Two current studies using email reminders to promote educational courses in the prevention of glucocorticoid-induced osteoporosis and in the secondary prevention of cardiovascular disease in patients following a myocardial infarction have been designed to deliver the intervention immediately following agreement to participate [13].

Findings from follow-up evaluation question data indicate that CME providers interested in targeting specific groups of physicians may benefit from using alternative methods of CME recruitment and delivery. CME providers targeting DOs may want to explore ways to engage DOs in web-based learning activities or consider focusing activities that target DOs to local or national meetings. Providers of CME who are interested in engaging male physicians and graduates of U.S. medical schools may find email reminders useful, but they may also want to explore additional methods of recruitment.

Persistent email reminders did increase physicians' response rates to online education, but response rate decreased with the number of reminders. The first three reminders produced the largest responses, with decline after the 10^th ^reminder. Based on our experience, it would seem reasonable that providers and researchers with limited resources consider focusing their announcements/reminders on the first 3–10 encounters. Data from time of log-on underscores the advantages of asynchronous online interventions for busy clinicians. Traditional "live" online symposia scheduled for the middle of the day might appeal to the physicians who logged on between 10 AM and 2 PM (10:00–14:00), but data from this intervention suggest that many physicians have more available time later in the day for online educational activity. The investigators findings of Monday being a frequent day for log-on was unexpected, but may offer an opportunity for future study designs to include weekend email broadcasting rather than a Thursday broadcast.

The topic of the online educational activity may influence response to email announcements and reminders. While baseline Chlamydia screening rates were relatively low in both groups, the significant difference between the groups may indicate that those who are likely to perform better according to clinical practice guidelines are more likely to participate in online educational strategies that reinforce their use. Or the higher screening rates may be associated with a higher degree of interest in the overall topic area of women's health.

Also related to the topic addressed in this online study, previous studies of preventive practices of female physicians have indicated they are more likely than males to promote preventive practices [14] and screening [15] including Chlamydia screening among their female patients than are male physicians. The advertised educational topic for this study was an online women's health course. More female physicians than male physicians responded to the email course reminders, but the topic may have had more appeal to female physicians than to male physicians, leaving the issue of whether there are gender differences among physicians in response to email reminders unresolved.

Comparisons of characteristics of participant physicians and non-participant recruited physicians may be useful to those designing online recruitment and engagement strategies for future studies. Those using email reminders to communicate with physician populations including large numbers of DOs, however, may benefit from considering blended methods of CME recruitment and delivery. Using various methods of reaching providers, may also enhance DO participation. CME providers targeting DOs may want to explore additional ways to engage DOs in web-based learning activities or consider focusing activities that target DOs at local or national meetings.

## Conclusions

Physicians' online clinical information seeking and engagement in online education continues to grow [12]. Researchers of online interventions who are attempting to improve the quality of healthcare and physician performance should continue to study and evaluate physician online behavior. Knowing when, where, and how physicians seek information on the Internet, and how they respond to receiving specific information pushed toward them, will prove to be very useful for targeting future quality improvement interventions.

Reminding physicians often via email about online educational opportunities appears to increase engagement in a community-based primary care physician audience. The early and consistent implementation of this push technology may increase physicians' utilization of interventions designed to improve practice.

## Authors' contributions

MA participated in the statistical analysis and drafted the manuscript. BCC participated in the statistical analysis and drafting of the manuscript. LC participated in all phases of the project. TW participated in the draft of the manuscript. CS participated in the design and coordination of the study and the drafting of the manuscript. MNR participated in the coordination of the study. NWW participated in the design and implementation of the study. JJA conceived of the study and participated in all phases of the study. All authors read and approved the final manuscript.

## Competing interests

The authors declare that they have no competing interests.

## Pre-publication history

The pre-publication history for this paper can be accessed here:


